# Quantifying the foodscape: A systematic review and meta-analysis of the validity of commercially available business data

**DOI:** 10.1371/journal.pone.0174417

**Published:** 2017-03-30

**Authors:** Alexandre Lebel, Madeleine I. G. Daepp, Jason P. Block, Renée Walker, Benoît Lalonde, Yan Kestens, S. V. Subramanian

**Affiliations:** 1 Evaluation Platform on Obesity Prevention, Quebec Heart and Lung Institute Research Centre, Quebec City (QC), Canada; 2 Graduate School of Urban Planning and Land Management, Laval University, Quebec City (QC), Canada; 3 Department of Urban Studies & Planning, Massachusetts Institute of Technology, Cambridge (MA), United States of America; 4 Department of Population Medicine, Harvard Medical School/Harvard Pilgrim Health Care Institute, Boston (MA), United States of America; 5 Zilber School of Public Health, University of Wisconsin, Milwaukee (WI), United States of America; 6 Social and Preventive Medicine Department, Université de Montréal, Montréal (QC), Canada; 7 Research Centre of Centre hospitalier de l’Université de Montréal, Montréal (QC), Canada; 8 Department of Social and Behavioral Sciences, Harvard School of Public Health, Boston (MA), United States of America; University of Tennessee Health Science Center, UNITED STATES

## Abstract

This paper reviews studies of the validity of commercially available business (CAB) data on food establishments (“the foodscape”), offering a meta-analysis of characteristics associated with CAB quality and a case study evaluating the performance of commonly-used validity indicators describing the foodscape. Existing validation studies report a broad range in CAB data quality, although most studies conclude that CAB quality is “moderate” to “substantial”. We conclude that current studies may underestimate the quality of CAB data. We recommend that future validation studies use density-adjusted and exposure measures to offer a more meaningful characterization of the relationship of data error with spatial exposure.

## Introduction

The influence of local food environments on dietary behaviors has generated much interest among researchers and policymakers concerned about lifestyle, obesity, and other chronic health conditions [1–6]. However, associations between measures of exposure to food establishments (e.g. access or availability) and health or health-related behaviours are mixed [7–11]. While some researchers have found positive associations between measures of food establishment exposure and health outcomes [12–15], several studies report negative associations [16, 17]. Errors in the information used to identify food establishments may contribute to the disparate nature of existing results [18].

Researchers seeking area-based measures of exposure to food establishments, commonly referred to as the “food environment” [10] or the “foodscape” [19, 20], often rely on commercially available business (CAB) data. CAB data are often more readily available than governmental resources (e.g. food establishment inspection or licensing records) and require less time to obtain than field observations, leading to their widespread use [12, 18, 21, 22]. Assessments of the validity of such data sources have increasingly been recognized as an important component of public health studies examining the food environment [23, 24]. These assessments compare CAB data sources characterizing retail food environments with a “gold standard” such as ground truthing–the systematic observation of the study area–or an official government listing (e.g. food safety inspection records).

A recent review of such studies found wide variability in CAB data validity estimates, ultimately recommending that researchers rely on primary data collection whenever possible [25]. Although this solution may be ideal, collecting primary data is often time consuming and expensive, and one could expect further use of CAB data in research along with validity measures. However, global measures of validity are sensitive to differences in the total count of food stores, while such absolute changes may not significantly affect relative food environment measures exposure (such as the number of stores per capita, or density-adjusted measurements) researchers ultimately use to explore associations of the foodscape and health [26, 27]. Furthermore, the traditional validation measures used are not necessarily comparable across studies since they are sensitive to sample size and dispersion measurement distribution [28]. These flaws arise as the validity measures evaluated, which were drawn from the field of epidemiology, are not designed to evaluate spatial exposure data [29, 30]. Counted data within a geographic area are known to be driven by the underlying urban density, and are not necessarily a relevant proxy to estimate a specific exposure in an epidemiologic study [31]. For example, where a great number of fast-food restaurants is found, a great number of other services, such as banks or pet shops, will also be found [32]. Drawing on approaches from geography, we argue that per capita measures are potentially more useful to estimate the exposure because they offer researchers an understanding of how errors in the CAB data affect measures of exposure to food outlets—and thus offer more insight on the likely effect on researchers’ ultimate outcome of interest: the association of the food environment and diet-related health [31].

The aim of this study was to characterize and interpret existing estimates of the validity of CAB data sets for foodscape research. The methodology includes three components: 1) a systematic review of studies assessing the validity of CAB food establishment data sources in public health and social epidemiology research, 2) a meta-analysis of the results obtained from these studies, and 3) a case study comparing the interpretation of validation measures with the correlation of density-adjusted food environment exposure between a CAB data source and a gold standard. Components (1) and (2) offer researchers a general estimation of the magnitude and type of error commonly observed in CAB data, while component (3) examines the effects error may have on research outcomes.

## Materials and methods

### Systematic review

The review focused on studies that investigated the validity of CAB food environment data sources. It was performed using PubMed, which gives access primarily to the MEDLINE database of public health and social epidemiology related scientific references. We created a two-step procedure for searches. We built two independent research “blocks” and then identified the manuscripts that were present in both blocks. Although the first block contained some “food outlet” terms, the search used search terms related to the types or descriptions of data sources that could be used to identify “food outlets” including, “commercial database”, “ground truthing”, “secondary commercial data”, and variations of these terms using the “OR” function. The second block included terms describing food establishments: “food supply”, “food stores”, “foodscape” OR “eating places”. The detailed search strategy is available in the supporting information document (S1 File- Search strategy) and presents all keywords used for each block.

**Fig 1:**
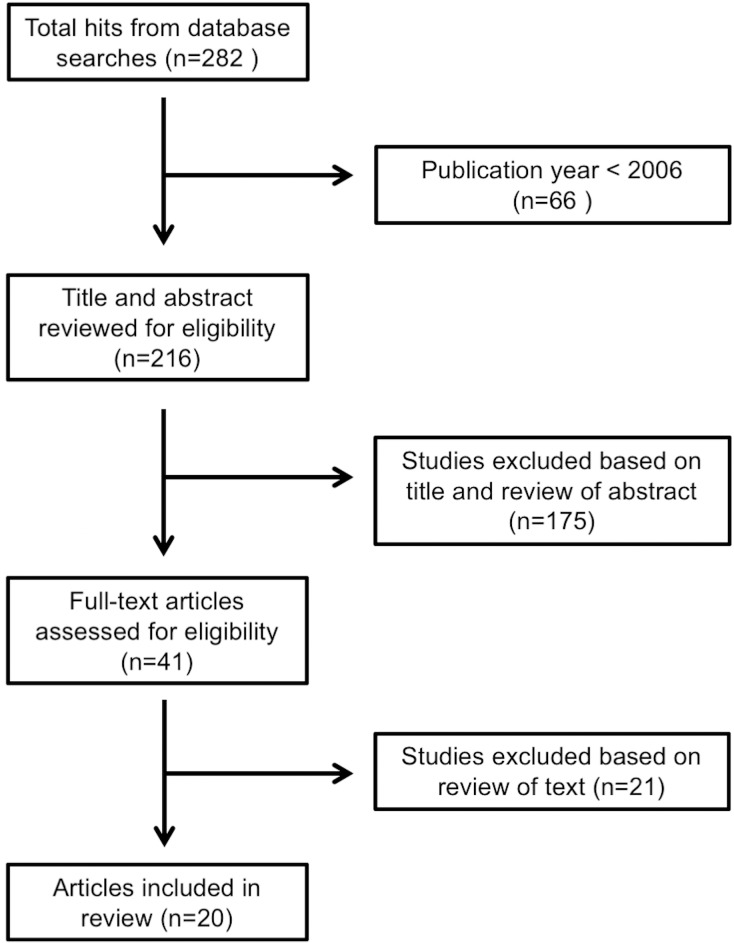
Flow chart of the search procedure.

The review was limited to primary studies published in English between January 1^st^ 2006 and June 30^th^ 2015, covering the last decade, where considerable progress has been made in GIS-based investigations [33]. Titles and abstracts were then examined by two researchers (BL, AL) to identify all studies that compared a CAB data source to a gold standard, such as primary data collection (e.g. ground truthing) or government lists (food establishment inspections or licensing records). For those titles and abstracts that did not reveal these criteria, two researchers examined the entire article (BL, MD) and two researchers checked the final selection (MD, AL). The search procedure was summarized in a flow chart (Fig 1). Examples of manuscripts that did not meet our inclusion criteria are listed in the supporting information document (S2 File- Examples not included).

All included studies reported epidemiologic validation measures to quantify error in the CAB datasets. These measures were typically constructed from the number of true positives, false positives, and false negatives (see Table 1). Authors used these measures to calculate sensitivity (the proportion of establishments in the gold standard also found in the CAB data source), positive predictive value (the proportion of establishments in the CAB data source also found in the gold standard) and concordance (the proportion of all establishments identified in the gold standard or CAB that are in both data sources, including true positives, false positives, and false negatives). Because most studies reported validation measurements across a variety of store types or between multiple CABs, we calculated the median and interquartile range of the measures reported in each study. We also examined whether these studies reported evidence of systematic bias according to the most commonly reported contextual measurements: neighbourhood socioeconomic status, population density, and neighbourhood racial composition. Each paper measured significance differently. As a result, we also relied on author interpretations to evaluate the results; details of author interpretations can be found in the supplementary documentation (S3 File- Author interpretations).

**Table 1:** Validity score measurements of a CAB dataset using a gold standard.

Gold Standard				Validity Score	
		*Present*	*Absent*	Sensitivity	TP/TP+FN
**CAB**	***Present***	True positive (TP)	False positive (FP)	Positive predictive value	TP/TP+FP
	***Absent***	False negative (FN)	True negative (TN)	Concordance	TP/TP+FP+FN

### Meta-analysis of CAB validity measures

The second component of this study, a meta-analysis of validity results, aimed to assess whether the use of classification schemes, characteristics of the CAB data source, or the sample size examined in the study were associated with error rates. To construct the meta-analysis, we followed several steps. First, one researcher (MD) extracted the concordance, positive predictive value (PPV), and sensitivity values across stores and CAB types from each reviewed study (S4 File- Meta-analysis dataset). For example, a study that validated both Dun & Bradstreet and InfoUSA data with ground-truthed food outlet locations for supermarkets, grocery stores, and fast food restaurants would have six entries for each concordance, PPV, and sensitivity category (separate for each CAB and each type of food establishment). Hereafter, we refer to these different types of food establishments as CAB subsamples and the multiple entries per subsamples as measures on CAB subsamples.

First, boxplots compared the distribution of sensitivity, PPV and concordance estimate across aggregated samples of all food outlets and across the subsamples to evaluate whether detailed store type classifications led researchers to report lower validity scores.

Next, we examined the associations between CAB characteristics and levels of validity. Studies commonly reported the geographic region for which the CAB was obtained as well as the CAB name. We used these data to construct scatterplots comparing subsample validity estimates with the sample size (defined below), stratified by country. Boxplots additionally compared the distributions of validity estimates for the most commonly examined CABs (InfoUSA and Dun & Bradstreet).

Finally, we examined the association of sample size and validity. We estimated the correlation of validity measurements of each CAB subsample with its sample size using Spearman’s rank correlation coefficient. Sample size was calculated as the number of food outlets of the type under examination that exist in the CAB, whenever available, or as the total unique outlets examined in either CAB or gold standard when CAB numbers alone were not reported.

### Case study comparing validity scores and correlation of per capita exposure

This case study analysis used data from Boston (Massachusetts, USA) to assess the relationships of commonly used validity measures and food outlet exposure per capita at the neighbourhood level, the type of measurement ultimately of interest in health and place research. InfoUSA food outlet data for 2009 (obtained through ESRI Business Analyst) was compared against the 2009 food store database maintained by the city of Boston’s Inspectional Services Department (ISD); the former dataset served as the CAB data, while the latter—a comprehensive, well-maintained and validated government data source—was treated as the gold standard. We considered the ISD data to be the gold standard because the city of Boston is required by law to license all food establishments and to conduct annual food safety inspections [34]. Food safety inspectors visit these fixed locations, and food establishments are required to obtain a permit to operate. Therefore, there is regular “ground truthing” by the government officials. Some establishments could be missed if they did not obtain proper permits, of if they mobile installations.

The InfoUSA data set included all business establishments located within 500m buffers of the study’s selected census tracts; North American Industry Classification System (NAICS) codes were used to identify and classify establishments selling food or beverages (n = 7465). Each store classification was reviewed and category assignments were revised according to keywords as well as researchers’ local area knowledge. In the ISD data, each entry was reviewed individually to remove duplicates and non-commercial entities (e.g. children’s feeding programs), and was categorized according to the NAICS codes definition. All establishments (n = 1581) except those without identifiable civic addresses (n = 40) were geocoded with ArcGIS 10.0; the coordinates for addresses that could not be geocoded (n = 4) were obtained from Google Maps and validated in the field.

The clean data sets were merged in ArcGIS 10.0 according to spatial location. Each unique food establishment was examined to determine the number of stores found only in the ISD data (false negatives), those found only in the InfoUSA data (false positives), and those found in both datasets (true positives). These counts were assessed across all food establishments—regardless of classification—as well as across each of four food outlet types: full-service restaurants, fast-food restaurants, caterers and grocery/convenience stores. We consider a listing to be in both data sources (a true positive) if an outlet with the same name was observed was very close (within +/- 200 m) and on the same street in both data sources. Sensitivity, PPV and concordance between the two data sets were calculated following the formula in Table 1.

In addition to the validity statistics describing the entire area, we computed the correlation between the per capita food environment exposure estimated by both data sets. We used Spearman’s rank correlation coefficient to account for the non-normal distribution of the data. For each Boston neighbourhood (n = 27), the number of stores per capita based on the estimated 2009 population in census tracts was calculated for both the InfoUSA data and the ISD data; the correlation between the per capita exposure across neighbourhoods was then calculated for all food establishments as well as for full-service restaurants, fast food restaurants, caterers, and grocery/convenience stores. In addition of showing the level consistency of both datasets, were compared the validation measurements (concordance, PPV and sensitivity) and the correlations of the per capita exposure estimations to reveal if these different validation indicators provided diverging assessments of CAB data quality.

## Results

### Systematic review

The systematic search strategy produced 20 manuscripts that validated at least one CAB data source in comparison with a gold standard (Table 2). Twelve studies were conducted in the United States [9, 35–45], four were conducted in the United Kingdom [19, 46–48], two were conducted in Canada [49, 50] and two were conducted in Denmark [51, 52]. Eight of these studies reported concordance between a gold standard and CAB data sources, 15 studies computed positive predictive values (PPV), and 16 computed the sensitivities; five studies reported all three validation indices. The median reported PPV (across all store type subsamples) was 77% (IQR = 30%), sensitivity 60% (IQR = 37%), and concordance 71% (IQR = 57%) across all studies.

**Table 2:** Summary information for 20 foodscape validation manuscripts, 2006–2015.

*1*^*st*^ *Author*	*Year*	*Area*	*Outlet Sample*	*PPV*		*Sensitivity*		*Concordance*		*Association with Area Characteristics*		
			*(n)*	*Med*	*IQR*	*Med*	*IQR*	*Med*	*IQR*	*SES*	*Density*	*Race*
***Wang***	2006	*USA*	357	0.26	-	0.72	-	-	-	-	-	-
***Paquet***	2008	*CA*	168 to 181	0.94	0.04	0.75	0.09	0.71	0.06	NS	-	-
***Cummins***	2009	*UK*	325	0.87	0.01	-	-	-	-	NS	-	-
***Bader***	2010	*USA*	89 to 1052	-	-	-	-	0.88	0.06	NS	*	NS
***Hosler***	2010	*USA*	5 to 107	0.89	0.18	0.54	0.66	-	-	-	-	-
***Jilcott***	2010	*USA*	1 to 432	-	-	-	-	0.91	0.19	-	-	-
***Lake***	2010	*UK*	393 to 564	0.82	0.06	0.52	0.04	-	-	-	-	-
***Liese***	2010	*USA*	7 to 1694	0.84	0.12	0.69	0.23	-	-	-	NS	-
***Longacre***	2011	*USA*	8 to 1340	-	-	-	-	0.39	0.07	-	*	-
***Powell***	2011	*USA*	101 to 2596	0.57	0.25	0.38	0.27	0.25	0.13	*	*	*
***Toft***	2011	*DK*	166	0.92	-	0.82	-	-	-	-	*	-
***Fleischhacker***	2012	*USA*	37 to 891	0.38	0.46	0.60	0.54	0.28	0.46	-	-	-
***Gustafson***	2012	*USA*	2 to 540	0.84	0.28	0.98	0.04	-	-	-	-	-
***Lake***	2012	*UK*	19 to 210	0.81	0.04	0.87	0.08	-	-	NS	NS	-
***Rossen***	2012	*USA*	169	-	-	0.77	0.08	-	-	NS	-	NS
***Svastisalee***	2012	*DK*	109 to 189	0.95	0.05	0.81	0.13	0.78	0.09	**-**	**-**	**-**
***Burgoine***	2013	*UK*	93 to 2100	0.76	0.06	0.61	0.19	0.50	0.16	NS	NS	-
***Clary***	2013	*CA*	1 to 410	0.67	0.18	0.56	0.13	-	-	NS	-	-
***Liese***	2013	*USA*	7 to 898	0.60	0.20	0.40	0.35	-	-	*	-	*
***Rummo***	2014	*USA*	0 to 228	-	-	0.55	0.46	-	-	-	-	NS

* This study reported a significant difference in CAB validity scores across neighbourhoods according to this area characteristic.

**NS** This study reported a non-significant differences in CAB validity scores across neighbourhoods according to this area characteristic.

Thirteen studies examined the relationship of CAB data source validity scores and neighbourhood characteristics. Seven of the nine studies that examined neighbourhood socioeconomic status and three of the five studies that examined race concluded that there were no significant differences in CAB validity across neighbourhoods. In contrast, four of the seven studies that examined population density did find evidence of systematic differences in validity according to commercial or population density. It should be noted, however, that many of these studies tested several associations across different subsets of the CAB data without correcting for multiple testing, and thus the results may be subject to an inflated type 1 error rate [53].

### Meta-analysis of CAB validity measures

A total of 540 measures on subsamples were extracted from the 20 studies under review. Sixteen studies reported sensitivity (n = 235), 15 studies reported PPV (n = 163), and 8 studies reported concordance.

**Fig 2:**
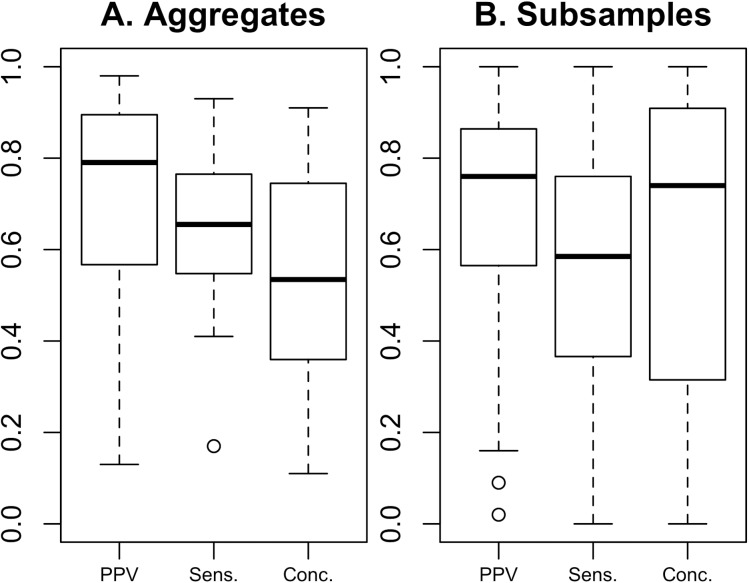
Median and interquartile range of validation measures across reviewed studies. A) Validity measures reported for aggregate store sample examined in the study (PPV n = 20; Sens n = 28; and Conc. n = 12). B) Validity measures reported for subsamples, defined as subsets of outlets examined according to outlet type (PPV n = 136; Sens n = 200; and Conc. n = 130). In each boxplot, the dark line indicates the overall median; in the case of 1B, the dark line is the median of the medians reported in studies. The upper and lower hinges of the box are the first and third quartiles, and the whiskers extend to approximately 1.5 times the interquartile range.

When aggregate samples were examined (Fig 2), studies reported slightly higher median PPV (79%) and sensitivity (66%) in contrast with the medians reported for examinations of subsamples (median PPV = 76%; sensitivity = 59%). Median concordance was slightly higher for the subsamples (74%) than for the (53%); however, subsample estimates had much higher variability (Fig 2B) than did aggregate sample estimates (Fig 2A).

**Fig 3:**
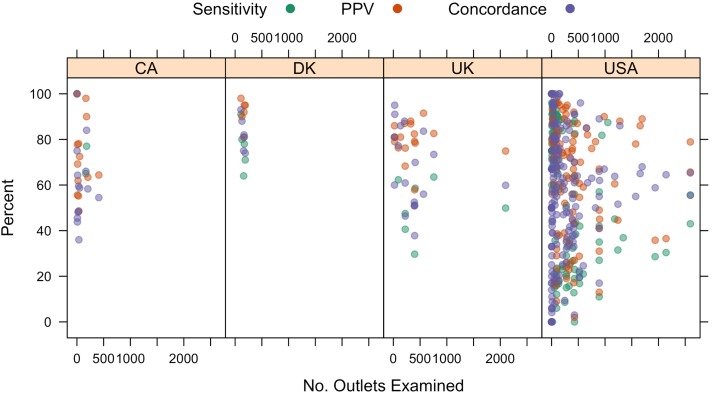
Validity estimates across countries. Points represent the estimate obtained for each validity measure, plotted against the number of outlets listed in the CAB dataset. There is a much smaller range of validity estimates obtained from the studies that have been conducted outside of the United States. However, we can also see that fewer studies have been conducted in Canada, Denmark, and the United Kingdom than in the United States. Furthermore, the studies that have been conducted outside of the United States have smaller sample sizes than many of the U.S. studies.

Between-country results (Fig 3) showed a greater range in PPV, sensitivity and concordance estimates in the United States as compared with other countries. Studies report comparable median validity scores in Canada (Sensitivity = 59%; PPV = 71%; Concordance = 71%) and the United Kingdom (Sensitivity = 61%; PPV = 81%; Concordance = 50%) to the medians reported for the US (Sensitivity = 59%; PPV = 75%; Concordance = 74%), although results from Denmark are consistently higher (Sensitivity = 82%; PPV = 94%; Concordance = 78%). However, there is a much smaller range of validity estimates obtained from the studies that have been conducted in Canada (Sensitivity IQR = 17%; PPV IQR = 25%; Concordance IQR = 6%), Denmark (Sensitivity IQR = 10%; PPV IQR = 5%; Concordance IQR = 9%), or the UK (Sensitivity IQR = 30%; PPV IQR = 8%; Concordance IQR = 16%) in contrast with the United States (Sensitivity IQR = 41%; PPV IQR = 35%; Concordance IQR = 61%)—though these differences in variability may be a product of the smaller numbers of studies or the smaller sample sizes for studies conducted outside of the U.S.

**Fig 4:**
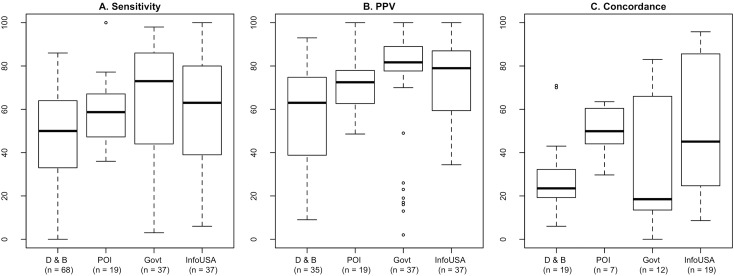
Validity estimates by dataset. Boxplots are used to compare the validity estimates from studies that assessed Dun & Bradstreet CAB, DMTI Spatial, Inc.’s Enhanced Points of Interest (POI) or UKPOI CABs, government datasets (e.g. from health registers, SNAP or WIC listings, store licenses, and tax registrations), and InfoUSA CAB.

In the comparison across different sources of CAB data, median validation scores tended to be lower in studies using Dun & Bradstreet datasets. However, all sources had a similar and wide range of validity measurements across studies, even among government data, and does not allow to clearly identify if a data source is more valid than the others (Fig 4).

The number of stores listed in the CAB was positively associated with sensitivity (Spearman’s Rank ρ = 0.178, p = 0.007) and inversely associated with PPV (Spearman’s Rank ρ = -0.287, p < 0.001) and concordance (Spearman’s Rank *ρ* = -0.646, p < 0.001), but this association was in part due to the presence of a very small number of stores examined. As an example in Table 3, when we examined the associations of validity measures and sample size while keeping CAB subsamples with a higher food store number (above 3, 10, and 30 observations), the strength, the sign and the p-value of the correlations changed importantly, suggesting the correlations were sensitive to the presence of subsamples having a small sample size in the distribution.

**Table 3:** Effect of small samples on Spearman’s rank correlation coefficient for sample size and validity measures.

Measure		All	n>3	n>10	n>30	n≤30
	*ρ*	0.178	-0.054	-0.210	-0.256	0.550
Sensitivity	p-value	0.007	0.460	0.006	0.002	<0.001
	n	235	187	170	150	74
	*ρ*	-0.288	-0.229	-0.182	-0.132	-0.604
PPV	p-value	<0.001	0.005	0.032	0.138	0.002
	n	163	148	140	128	24
	*ρ*	-0.646	-0.560	-0.495	-0.302	-0.294
Concordance	p-value	<0.001	<0.001	<0.001	0.003	0.041
	n	142	125	111	93	49

### Case study comparing validity scores and correlation of per capita exposure

The mean food store density per 1000 people, estimated for the 27 neighbourhoods of Boston, varied between the InfoUSA and the ISD datasets (Table 4). Both datasets had a very high standard deviation, which limited our ability to demonstrate significant differences either for all food stores or between each food store types. The validity estimates obtained for the 2009 Boston foodscape (Table 5) were comparable to those observed across the studies surveyed (Table 2). For all food stores, InfoUSA had sensitivity of 68%, PPV of 51%, and concordance of 41%. According to the Landis scale (<0.00 poor, 0.00–0.20 slight, 0.21–0.40 fair, 0.41–0.60 moderate, 0.61–0.80 substantial, and 0.81–1.00 almost perfect reliability) [30], which was used to interpret validity scores in a CAB related literature review [25], the dataset sensitivity would qualify as substantially reliable, while PPV and concordance would be considered as moderately reliable.

**Table 4:** Descriptive statistics of food store exposure per 1,000 residents in 27 neighbourhoods of Boston, MA.

	Population		All Stores		Fast Food		Full Service		Grocery		Caterer	
	Mean	SD	Mean	SD	Mean	SD	Mean	SD	Mean	SD	Mean	SD
**Boston ISD**	40591	65004	1.07	1.01	0.31	0.41	0.25	0.41	0.5	0.28	0.01	0.02
**InfoUSA**			1.61	1.14	0.30	0.39	0.46	0.67	0.37	0.2	0.02	0.03

**Table 5:** InfoUSA’s validity scores and correlation of per capita food store exposure as compared to Boston ISD, 2009.

Classification		Store Count			Validity score		Correlation
ALL FOOD STORES			**Boston ISD**				
	**InfoUSA**		**Present**	**Absent**	Sensitivity	0.68	
		***Present***	1572	1485	PPV	0.51	*ρ* = 86.9%
		***Absent***	741	N/A	Concordance	0.41	
FULL SERVICE RESTAURANTS			**Boston ISD**				
	**InfoUSA**		**Present**	**Absent**	Sensitivity	0.72	
		***Present***	587	818	PPV	0.42	*ρ* = 99.6%
		***Absent***	227	N/A	Concordance	0.36	
FAST FOOD RESTAURANTS			**Boston ISD**				
	**InfoUSA**		**Present**	**Absent**	Sensitivity	0.73	
		***Present***	566	221	PPV	0.72	*ρ* = 96.8%
		***Absent***	206	N/A	Concordance	0.57	
GROCERY & CONVENIENCE STORES			**Boston ISD**				
	**InfoUSA**		**Present**	**Absent**	Sensitivity	0.58	
		***Present***	413	396	PPV	0.51	*ρ* = 83.5%
		***Absent***	296	N/A	Concordance	0.37	
CATERERS			**Boston ISD**				
	**InfoUSA**		**Present**	**Absent**	Sensitivity	0.33	
		***Present***	6	50	PPV	0.11	*ρ* = 76.9%
		***Absent***	12	N/A	Concordance	0.09	

In contrast with the validity estimates, the relative food store exposure by neighbourhood—calculated as the number of stores available per capita in each neighbourhood—was similar between the two datasets (Table 5). The correlation of exposure to food stores per 1000 people between the gold standard (ISD) and the CAB (InfoUSA) was 86.9%. For each food store category, the correlations were 99.6% for full-service restaurants, 96.8% for fast-food restaurants, 83.5% for grocery and convenience stores, and 76.9% for caterers. All correlations were significant at the 1% level.

## Discussion

Public health authorities and researchers are increasingly seeking to estimate the association of the food environment with health outcomes or diet, but the quality of food environment data poses a significant challenge. The main purpose of this research was to analyse studies assessing the validity of commercially available business (CAB) data sources for food establishments in order to characterize and interpret the validation indicators commonly used in health and place studies. This study consists of three main components: 1) a description of CAB performance across studies, 2) a meta-analysis of the associations of data errors with area characteristics, and 3) a critique of the interpretation of validity measures through an alternative method of validating geographic data.

### Between study CAB performance

The quality of CAB food outlet databases has been the subject of at least twenty studies to date. The reviewed studies used the epidemiological validation measures of sensitivity, positive predictive value and concordance to assess the data quality. The resulting measures showed a high variability, but the majority of sensitivity and PPV results fall between 40% and 85%. Applying the interpretations of the Landis Scale, the above-mentioned results can be seen as moderate to substantial reliability. However, the Landis Scale was originally designed to evaluate Kappa statistics, which are slightly different from the validity measures surveyed in this study (Munoz and Bangdiwala 1997). The Kappa statistic is a measure of precision between raters that compares the observed agreement between two sources with the agreement that would occur by chance; in contrast, sensitivity, PPV and concordance are not adjusted for random agreement (cite: Viera & Garrett 2005), and thus its levels deserve a stricter interpretation. Furthermore, as Landis and Koch noted, the scale’s statistical thresholds were not supported by empirical investigations, but rather provided a useful benchmark for a discussion (Landis and Koch 1977). Furthermore, it is important to mention that several CAB validation studies directly referred to an interpretation scale proposed by Paquet [54] to analyze the concordance of their observations, which in turn referred to Janse [55]. The latter is actually a meta-analysis of patient-doctor agreement on the quality of life, and provide no justification to interpret the degree of agreement. Analysing the concordance between CAB databases is a very different research context and may not be directly transferable. The validity of a CAB would be better evaluated in terms of the error’s likely effect on study outcomes. For example, if 20% of fast food outlets are incorrectly classified in the CAB, will associations of fast food outlet exposure and diet-related health be compromised? Not necessarily, because one type of food outlet may be replaced by a similar type of establishment. In this situation, the validity measure will go down, while the exposure to food outlet of similar type would stay about the same. As only one study has examined the effect of dataset error on measurements of the food environment [56] and no study, to our knowledge, has examined the effect of data set error on study outcomes, this question remains unanswered. Future research could address this gap through methods similar to those presented in this paper’s case study—i.e. through field research that, in addition to calculating validity scores, also examines the correlations between food environment exposure measures constructed from secondary and from gold-standard data—or through simulation studies that estimate the potential effects of various levels of error on measures of food environment exposure.

This study found a statistically significant relationship between sample size and validity measures. However, the association of sensitivity and CAB sample size reversed direction when subsamples with very few listings—and thus with extreme values—were excluded. Although the associations of PPV and concordance were negative and statistically significant both for all subsamples and for subsamples with large n, excluding subsamples with few listings led to a large decrease in the magnitude of the association. As a result, comparing validity statistics between studies with large differences in the number of observations appears highly questionable, and we recommend that researchers use caution when interpreting data disaggregated into very small subcategories.

This study did not find evidence of noteworthy differences in quality across different CABs. This finding does not endorse those reported in a recent review, which reported high levels of agreement in InfoUSA and government data in comparison with other secondary data sources [25]. Although we also observe that these two sources had a slightly higher median validity measurements, there is strong variability around the median values, preventing a clear conclusion regarding each CABs relative reliability.

Comparability between countries is also limited. There is some evidence that studies conducted in Denmark, Canada, and the United Kingdom obtained higher validity measures than those conducted in the United States, but studies in the former three countries have been much fewer in number and used smaller samples than many of the studies conducted in the U.S.

### Associations with area characteristics

This meta-analysis did not reveal evidence of a systematic relationship between CAB error and neighbourhood characteristics such as socioeconomic status or neighbourhood racial composition. Of the nine studies that disaggregated measures by neighbourhood socioeconomic status, only two reported a relationship with validity measures, and three of the five studies that examined racial demographics found no significant association with CAB data validity. These results align with the measures reported in a recent, similar review [25].

Among studies using CAB data in areas with variability in commercial or population density, four out of seven studies found that validity measures differed significantly between areas with high versus low densities. This result is possibly linked to the number of food stores under investigation as we demonstrated previously, and where the smallest samples (n<3) tended to lead to extreme validity scores. This finding suggests that validity scores are highly sensitive to very small sample size and thus may offer limited insight for studies conducted in rural areas or studies that disaggregate outlet data into many food outlet categories.

### Comparison of validity indicators with a measure of exposure

This paper used a case study from Boston (Massachusetts, USA) to compare the validity measures with a more common characterization of spatial exposure data, correlation of per capita exposure. While the three validity scores identified many errors in the CAB data, the per capita exposure to the foodscape was highly correlated between the CAB and gold standard data sources. The validity measures, originally developed to evaluate the quality of diagnostic tests, may not be suited to the measurement of spatial exposure data. The calculation of true positives, false positives, and false negatives requires that the outlet characteristics in the CAB data be nearly identical to those in the gold standard dataset. Many studies did consider listings with slight errors (e.g. incorrect names but correct classifications) as true positives, but minor errors in address or classification would have been listed as false positives, while their corresponding “real-world” outlet would be considered a false negative. Small errors can thus lead to large differences in validity measures despite a high level of similarity between *per capita exposure* to CAB food outlets and to gold standard food outlets.

### Strengths and limitations

This study is, to our knowledge, the first study to compare estimates of food environment dataset validity across countries; our assessment of the association between validity scores and sample sizes also offers researchers insight on the effects of detailed store classification schemes. However, this study did not test for associations between study characteristics (e.g. funding sources or research design) and CAB validity scores. The high variance observed in estimates of sensitivity, specificity, and positive predictive value thus may reflect differences in the quality of the studies examined rather than true differences in dataset quality. It should also be noted that this review relied only on data extracted from published studies. We did pursue unpublished data; thus the results may be affected by publication bias.

Although exposure measurements would allow a better assessment of the food environment, they also have limitations. The computation of a relative indicator, such as per-capita measures, is clearly pertinent for between-area or between-study comparison analyses, but it is dependent on the geography on which it is computed (e.g. the size and the borders of a neighbourhood) [57]. Also, correlation may not be the best validation tool when the objective is to construct measures of access to food sources (e.g. measuring the closest fast-food restaurant from home, or the mean distance to the three closest convenience stores) for which the precision of the geographic information is particularly important.

## Conclusions

All studies inspected here examined global error in preliminary food environments data. Further research is needed to understand how error affects the food environment measurements that are ultimately used in health and place research, but this work can offer guidance for future validation studies.

Although the majority of CAB data sources have moderate to substantial reliability according to the Landis scale, this scale may not provide adequate guidance to evaluate CAB validity. No guidelines currently exist to interpret validity measures specifically for geocoded built environment databases and their interpretation requires caution. We thus suggest that the analysis of validity measures should be accompanied by relative measure of exposure. Researchers should further be cautious in disaggregating data by outlet classification and geography as the use of data subsets with very small sample sizes can lead to the proliferation of extreme results. The results of the case study in Boston brought new insight on this aspect, suggesting that existing validation studies may underestimate the quality of CAB data sources for food environments research. Although validity measures indicated substantial errors between the CAB and the gold standard, when adjusted for neighbourhood population density (i.e. per capita exposure to foodscape), a relatively high correlation was found between both datasets. Future studies should include measures that better evaluate the effective of error on spatial exposure—correlation or “representativity” [50]—to offer a more meaningful characterization of CAB data quality when the aim is to estimate the exposure to the food environment.

While the evidence, presented in this study, of a high correlation in measures of per capita exposure obtained from CAB and gold standard data sets will be reassuring to researchers, the results are less promising for practitioners. A policymaker who prohibits fast food restaurants from locating within a set distance of schools, for example, will need exact data on outlet locations; the lower levels of validity observed in our systematic review suggest that policies requiring exact information on store locations will need to be accompanied by improved data collection mechanisms.

Although all CAB datasets include error, the systematic underestimation of CAB data validity may be leading researchers to conduct time- and cost-intensive primary data collection efforts that ultimately lead to little improvement in the research quality. Such primary data collection may be necessary in the case of a study area with high variability in population density, but food environment validation research does not offer evidence of systematic error in relation to race or socioeconomic deprivation. Further research should be conducted to develop validity measurements adapted for geographic data and to quantify the effect of data set error on measures of exposure.

## Supporting information

S1 File Search strategy. (DOCX)

S2 File Example not included. (DOCX)

S3 File Author interpretation. (DOCX)

S4 File Meta-analysis dataset. (ZIP)

S5 File PRISMA Checklist. (DOC)
