# Supplementary material for: Quantifying the foodscape: A systematic review and meta-analysis of the validity of commercially available business data
Source: PLoS One. 2017 Mar 30;12(3):e0174417. doi: 10.1371/journal.pone.0174417 (PMC5373546; doi:10.1371/journal.pone.0174417)
Supplement: S1 File — (DOCX) [file pone.0174417.s001.docx]

S1- Search strategy

The search strategy for block 1 was performed in PubMed:

*"Secondary datasets"[TIAB] OR "secondary data"[TIAB] OR "Databases, Factual"[Mesh:NoExp] OR "Directories as Topic"[Mesh] OR "directory data sources"[TIAB] OR "Geographic Information Systems"[TIAB] OR "Food Outlet Databases"[TIAB] OR "Ground truthing"[TIAB] OR "Remote Sensing Technology"[Mesh] OR "secondary sources"[TIAB] OR "existing data Sources"[TIAB] OR "commercial database"[TIAB] OR "Commercial data sources"[TIAB] OR "secondary commercial data"[TIAB] OR "historical data"[TIAB] OR "Data Collection"[Mesh:NoExp] OR "food outlet location data"[TIAB] OR "data collection"[TIAB]*

The search strategy for block 2 was performed in PubMed:

*"Food Supply"[Majr] OR "food environment"[TIAB] OR "food stores"[TIAB] OR Foodscape[TIAB] OR "retail food stores"[TIAB] OR "Food outlets"[TIAB] OR "eating places"[TIAB] OR "food sales places"[TIAB] OR "fast food access"[TIAB] OR "food access"[TIAB] OR "fast food outlets"[TIAB] OR "Fast Foods/supply and distribution"[Majr]*

A total of 282 manuscripts were found in both blocks.
