# Supplementary material for: Quantifying the foodscape: A systematic review and meta-analysis of the validity of commercially available business data
Source: PLoS One. 2017 Mar 30;12(3):e0174417. doi: 10.1371/journal.pone.0174417 (PMC5373546; doi:10.1371/journal.pone.0174417)
Supplement: S2 File — (DOCX) [file pone.0174417.s002.docx]

S2- Examples of documents not included

Before 2006 (n=66):

1. Horwath CC, Worsley A. Assessment of the validity of a food frequency questionnaire as a measure of food use by comparison with direct observation of domestic food stores. American journal of epidemiology. 1990 Jun;131(6):1059-67. PubMed PMID: 2343858. Epub 1990/06/01. eng.

2. Aranda-Pastor J, Menchu MT, Kevany JP. [Operational evaluation of a project of food-nutrition surveillance]. Archivos latinoamericanos de nutricion. 1981 Sep;31(3):439-59. PubMed PMID: 7344638. Epub 1981/09/01. Evaluacion operacional de un proyecto de vigilancia alimentaria--nutricional. spa.

3. Beaton GH. Perspectives and priorities in food and nutrition planning. Basic life sciences. 1976;7:37-44. PubMed PMID: 782431. Epub 1976/01/01. eng.

4. Huppert FA, Van Niekerk JK. Dehydroepiandrosterone (DHEA) supplementation for cognitive function. The Cochrane database of systematic reviews. 2001 (2):CD000304. PubMed PMID: 11405958. Epub 2001/06/19. eng.

5. Bhattacharya J, Currie J, Haider S. Poverty, food insecurity, and nutritional outcomes in children and adults. Journal of health economics. 2004 Jul;23(4):839-62. PubMed PMID: 15587700. Epub 2004/12/14. eng.

6. Robertson C, Best N, Diamond J, Elliott P. Tracing ingestion of 'novel' foods in UK diets for possible health surveillance--a feasibility study. Public health nutrition. 2004 Apr;7(2):345-52. PubMed PMID: 15003143. Epub 2004/03/09. eng.

Title and abstract screening (n=175):

1. Lesser LI, Hunnes DE, Reyes P, Arab L, Ryan GW, Brook RH, et al. Assessment of food offerings and marketing strategies in the food-service venues at California Children's Hospitals. Academic pediatrics. 2012 Jan-Feb;12(1):62-7. PubMed PMID: 22136808. Epub 2011/12/06. eng.

2. Lee JS. Food insecurity and healthcare costs: research strategies using local, state, and national data sources for older adults. Advances in nutrition (Bethesda, Md). 2013 Jan;4(1):42-50. PubMed PMID: 23319122. Pubmed Central PMCID: PMC3648738. Epub 2013/01/16. eng.

3. Shannon K, Kerr T, Milloy MJ, Anema A, Zhang R, Montaner JS, et al. Severe food insecurity is associated with elevated unprotected sex among HIV-seropositive injection drug users independent of HAART use. AIDS (London, England). 2011 Oct 23;25(16):2037-42. PubMed PMID: 21811140. Pubmed Central PMCID: PMC3956106. Epub 2011/08/04. eng.

4. Blasbalg TL, Hibbeln JR, Ramsden CE, Majchrzak SF, Rawlings RR. Changes in consumption of omega-3 and omega-6 fatty acids in the United States during the 20th century. The American journal of clinical nutrition. 2011 May;93(5):950-62. PubMed PMID: 21367944. Pubmed Central PMCID: PMC3076650. Epub 2011/03/04. eng.

5. Bader MD, Ailshire JA, Morenoff JD, House JS. Measurement of the local food environment: a comparison of existing data sources. American journal of epidemiology. 2010 Mar 1;171(5):609-17. PubMed PMID: 20123688. Pubmed Central PMCID: PMC2842213. Epub 2010/02/04. eng.

6. Moore LV, Diez Roux AV, Nettleton JA, Jacobs DR, Jr. Associations of the local food environment with diet quality--a comparison of assessments based on surveys and geographic information systems: the multi-ethnic study of atherosclerosis. American journal of epidemiology. 2008 Apr 15;167(8):917-24. PubMed PMID: 18304960. Pubmed Central PMCID: PMC2587217. Epub 2008/02/29. eng.

Full text screening (n=17):

1. Moore LV, Diez Roux AV, Franco M. Measuring availability of healthy foods: agreement between directly measured and self-reported data. American journal of epidemiology. 2012 May 15;175(10):1037-44. PubMed PMID: 22273535. Pubmed Central PMCID: PMC3353136. Epub 2012/01/26. eng.

2. Ohri-Vachaspati P, Martinez D, Yedidia MJ, Petlick N. Improving data accuracy of commercial food outlet databases. American journal of health promotion : AJHP. 2011 Nov-Dec;26(2):116-22. PubMed PMID: 22040393. Epub 2011/11/02. eng.

3. Glanz K, Sallis JF, Saelens BE, Frank LD. Nutrition Environment Measures Survey in stores (NEMS-S): development and evaluation. American journal of preventive medicine. 2007 Apr;32(4):282-9. PubMed PMID: 17383559. Epub 2007/03/27. eng.

4. Saelens BE, Glanz K, Sallis JF, Frank LD. Nutrition Environment Measures Study in restaurants (NEMS-R): development and evaluation. American journal of preventive medicine. 2007 Apr;32(4):273-81. PubMed PMID: 17383558. Epub 2007/03/27. eng.

5. Burgoine T. Collecting accurate secondary foodscape data. A reflection on the trials and tribulations. Appetite. 2010 Dec;55(3):522-7. PubMed PMID: 20832436. Epub 2010/09/14. eng.

6. Auchincloss AH, Moore KA, Moore LV, Diez Roux AV. Improving retrospective characterization of the food environment for a large region in the United States during a historic time period. Health & place. 2012 Nov;18(6):1341-7. PubMed PMID: 22883050. Pubmed Central PMCID: PMC3501601. Epub 2012/08/14. eng.
