# Supplementary material for: Quantifying the foodscape: A systematic review and meta-analysis of the validity of commercially available business data
Source: PLoS One. 2017 Mar 30;12(3):e0174417. doi: 10.1371/journal.pone.0174417 (PMC5373546; doi:10.1371/journal.pone.0174417)
Supplement: S3 File — (DOCX) [file pone.0174417.s003.docx]

S3- Author’s interpretation

| Wang 2006 | - No associations tested |
| --- | --- |
| Paquet 2008 | - “For both lists, the positive predictive value did not differ across SES tertiles or language groups (Fisher’s p>0.124).” |
| Cummins 2009 | - No significant difference in agreement level across deprivation tertiles. |
| Bader 2010 | - “We did not find a consistent pattern of association between socio-demographic characteristics and disagreement that would lead us to conclude that one data source consistently differs from the other in certain types of neighbourhoods. The only consistent predictor of disagreement was distance to the Loop [a proxy for commercial density]; disagreement was less likely in blocks further away from the center of the city.” |
| Hosler 2010 | - No associations tested |
| Jilcott 2010 | - No associations tested |
| Lake 2010 | - No associations tested |
| Liese 2010 | - “For stores, there were no marked differences between levels of urbanization in any of the 3 databases or the combined D&B and InfoUSA databases. A similar picture emerged for restaurants, the exception being significantly higher sensitivity in urban areas in the D&B data. We additionally evaluated the potential influence of tract racial composition or poverty on the validity estimates but found no evidence for any systematic differences (data not shown).” |
| Longacre 2011 | - “Public data were significantly less accurate for low population towns (Figure 2, P>0.001).” |
| Powell 2011 | - “Agreement for supermarkets and grocery stores did not differ significantly across tracts of different income levels for InfoUSA, but was higher in low- compared to middle income tracts in D&B. Agreement for convenience stores did not differ by tract SES in D&B but was lower in low- versus middle income tracts in InfoUSA.”   - “Among the low income tracts, some significant differences in agreement were observed by race and ethnicity.” - “By urbanicity, the results in Table 5 show that agreement in D&B for supermarkets, grocery stores and convenience stores was roughly the same in rural compared to either urban or suburban areas but higher in suburban versus urban areas. There were no significant differences for supermarket and grocery store agreement by urbanicity in InfoUSA, whereas for convenience store agreement was significantly lower in rural areas. The results also show that agreement for fast food restaurants was significantly lower in rural areas compared to urban or suburban tracts.” |
| Toft 2011 | - “…The sensitivity was found to be highest in the Copenhagen metropolitan area (84% vs. 76%) whereas the PPV was highest in the area outside Copenhagen (94% vs. 90%).” |
| Fleischhacker 2012 | - No associations tested |
| Gustafson 2012 | - No associations tested |
| Lake 2012 | - “The sensitivities…were considered to be ‘moderate’ to ‘excellent’ in all study areas.” - Table: PPV was similar in urban versus rural; sensitivities were generally similar – with a (low) outlier in the rural, low SES area. |
| Rossen 2012 | - “There were no differences between verified and unverified food outlets across a number of census tract-level SES variables and racial composition. However, unverified outlets were more likely to be located in census tracts with a higher number of vacant housing units." |
| Svatisalee 2012 | - No associations tested |
| Burgoine 2013 | - “Importantly, PPV’s across socio-economic and urban/rural divides were similar, both to each other, and to the statistic for all outlets. For sensitivity and percentage agreement, there were exceptions, including significantly better estimates of both in some more deprived quintiles, although no evidence of a trend existed, and in urban areas.” - Researchers “do not believe [a substantial difference across SES or urban/rural divides] was the case here.” |
| Clary 2013 | - “No significant difference was observed by CT characteristic (SES and language) for both *traditional* and *relaxed* measures.” |
| Liese 2013 | - “…For Dun and Bradstreet, no evidence for systematic differences in the validity statistics of supermarkets and grocery stores was found between levels of neighbourhood characteristics.” - Did find differences by race, income and poverty for InfoUSA (in 6 out of 18 tested associations). |
| Rummo 2014 | - “Agreement between the databases and the field census of food stores and restaurants did not differ substantially by Hispanic composition of census tracts.” |
