# Supplementary material for: Quantifying the foodscape: A systematic review and meta-analysis of the validity of commercially available business data
Source: PLoS One. 2017 Mar 30;12(3):e0174417. doi: 10.1371/journal.pone.0174417 (PMC5373546; doi:10.1371/journal.pone.0174417)
Supplement: S4 File — (ZIP) [file pone.0174417.s004.zip › S4-Meta-analysi_dataset/ReadMe.rtf]

ReadMe.txtThis folder contains the code and data for the study “Quantifying the foodscape: A systematic review and meta-analysis of the validity of commercially available business data” (Lebel et al., in submission to PLOS ONE). The data provided here are adequate to replicate Tables 2 and 3 as well as Figures 2, 3 and 4.The comparison of CAB and municipal data for the city of Boston, MA relied on proprietary data and thus cannot be made available to the public. Researchers hoping to replicate Tables 4 and 5 will need to obtain data from InfoUSA as well the the Boston Inspectional Services Department.Raw Data File: LEBEL_CAB_medians_20161127.csvClean Code File: LEVEL_ValidationCode_20161127.RPlease contact mdaepp@mit.edu with any questions
